# Aerobic and Anaerobic Methanotrophic Communities Associated with Methane Hydrates Exposed on the Seafloor: A High-Pressure Sampling and Stable Isotope-Incubation Experiment

**DOI:** 10.3389/fmicb.2017.02569

**Published:** 2017-12-19

**Authors:** David H. Case, Akira Ijiri, Yuki Morono, Patricia Tavormina, Victoria J. Orphan, Fumio Inagaki

**Affiliations:** ^1^Division of Geological and Planetary Sciences, California Institute of Technology, Pasadena, CA, United States; ^2^Geomicrobiology Group, Kochi Institute for Core Sample Research, Japan Agency for Marine-Earth Science and Technology, Nankoku, Japan; ^3^Geobiotechnology Group, Research and Development Center for Submarine Resources, Japan Agency for Marine-Earth Science and Technology, Yokosuka, Japan; ^4^Research and Development Center for Ocean Drilling Science, Japan Agency for Marine-Earth Science and Technology, Yokohama, Japan

**Keywords:** methanotrophs, high pressure incubation, methane hydrate, stable isotope probing, marine sediment

## Abstract

High-pressure (HP) environments represent the largest volumetric majority of habitable space for microorganisms on the planet, including the deep-sea and subsurface biosphere. However, the importance of pressure as an environmental variable affecting deep microbial life and their biogeochemical functions in carbon cycling still remains poorly understood. Here, we designed a new high-volume HP-sediment core sampler that is deployable on the payload of a remotely operated vehicle and can maintain *in situ* HP conditions throughout multi-month enrichment incubations including daily amendments with liquid media and gases and daily effluent sampling for geochemical or microbiological analysis. Using the HP core device, we incubated sediment and overlying water associated with methane hydrate-exposed on the seafloor of the Joetsu Knoll, Japan, at 10 MPa and 4°C for 45 days in the laboratory. Diversity analyses based on 16S rRNA and methane-related functional genes, as well as carbon isotopic analysis of methane and bicarbonate, indicated the stimulation of both aerobic and anaerobic methanotrophy driven by members of the *Methylococcales*, and ANME, respectively: i.e., aerobic methanotrophy was observed upon addition of oxygen whereas anaerobic processes subsequently occurred after oxygen consumption. These laboratory-measured rates at 10 MPa were generally in agreement with previously reported rates of methane oxidation in other oceanographic locations.

## Introduction

The deep-sea and the underlying marine sediment and rock represent the most volumetrically abundant habitats on the planet for microorganisms, where *in situ* pressure increases with depth. However, our understanding of the diversity, physiology, and adaptability of high pressure (HP)-tolerant (piezotolerant), HP-preferring (piezophilic), and HP-requiring (obligately piezophilic) microorganisms remains limited. The first active microbial communities from deep-sea sediments were described in 1957 from >10,000 meters below sea level (mbsl) in the Philippine Trench (Zobell and Morita, 1957). The first isolation of an obligate piezophilic species from deep-sea sediments did not occur until over 20 years later, which was a gammaproteobacterial *Colwellia* species (Yayanos et al., 1981). Since then, driven by the widespread use of molecular techniques, the diversity of piezotolerant and (obligately) piezophilic microorganisms has been extended to many clades of Bacteria (Yanagibayashi et al., 1999; Kato et al., 2008; Nagata et al., 2010; Zhang et al., 2015) and Archaea (Zeng et al., 2009; Birrien et al., 2011; Zhang et al., 2015), and probably varies significantly depending on the particular environmental setting.

Preliminary experiments have shown sediment-hosted microbial communities from the deep-sea habitat to be sensitive to changes in pressure: diversity, as measured by 16S rRNA genes, diverges over time if deep-sea sediments are maintained at atmospheric vs. at representative hydrostatic pressures (Yanagibayashi et al., 1999). Unsurprisingly, then, metabolic activity also differs whether experiments on deep-sea sediments are conducted at low or high pressures (Picard and Ferdelman, 2012). The sulfate-coupled anaerobic oxidation of methane (AOM), a major microbial metabolism worldwide in marine sediments, has been observed to proceed ~5-fold faster during *in vitro* laboratory incubations when pressures mimic ~100 m water depth (1.1 MPa) vs. atmospheric pressure (0.1 MPa; Nauhaus et al., 2002). Similarly, higher rates of sulfate reduction correlated with higher pressure in a study of Guaymas Basin hydrothermal sediments, with pressure up to 45 MPa (~4,500 mbsl) tested (Kallmeyer and Boetius, 2004). Physical and physiological adaptations of microorganisms to high-pressure might include alterations to fatty acid synthesis, membrane protein structure, ribosomal structure/assembly, methods of motility, and other as-yet unidentified modifications (Bothun et al., 2004; Simonato et al., 2006; Kato et al., 2007). Recently, piezophilic bacteria were isolated from ~2 km-deep subseafloor sediments, which were buried in energy-limited deep sedimentary environment and most likely persisted as spores over geologic time (Inagaki et al., 2015; Fang et al., 2017).

A significant obstacle in the study of piezophilic microorganisms has been sample recovery. High-pressure (e.g., deep-sea and subsurface) environments are generally difficult and expensive to access, and even once accessed it is challenging to maintain samples at HP during transport back to a research vessel and/or the home laboratory. Because of these logistical challenges, development of new sampling technology has been identified as a top priority in the field of HP-microbiology (e.g., Kim and Kato, 2010; Kato, 2011; Zhang et al., 2011, 2015). Development of deep-subsurface coring technologies which can maintain *in situ* pressure through recovery represents good progress, but can only be deployed on large drilling vessel such as the *JOIDES Resolution* or *Chikyu* at great expense and time commitment (Parkes et al., 2009; Kubo et al., 2014). Development of an affordable HP-sampling and experimental device that could be deployed on the payload of a piloted or remotely operated vehicle (ROV), which could retain *in situ* pressure through sample retrieval and shipment back to onshore laboratories would enable many members of the scientific community to pursue environmental microbiology research at high pressures. A device meeting many of these criteria was developed at the Japan Agency for Marine-Earth Science and Technology (JAMSTEC) in the 1990s, and successfully deployed, but was limited to small volumes of surface sediments (<50 mL) and was specifically designed to perform dilution-to-extinction experiments rather than stable isotope-probing or amendment incubations at high pressure in the laboratory (Kyo et al., 1991; Kato et al., 2008).

In order to address the technical considerations of working in deep-sea HP-environments, we designed, manufactured, and tested a new HP-core sampler (hereafter, “HP-Core”). The goal for this device was to be (i) deployable on the payload of an ROV, (ii) to have a “push core-like” structure enabling sampling down to >10 cm below seafloor, (iii) to maintain HP through recovery onboard ship and shipment to onshore laboratories, (iv) to have inlet ports for adding liquid media and/or gas phase (including stable isotope) amendments to the incubation chamber, and (v) to have an outlet port to enable time-course tracking of an experiment without sacrificing pressure on the entire vessel. After fabrication, deployment of the new HP-sampling device was tested on deep-sea sediments at the Joetsu Knoll, Japan, during the JAMSTEC research vessel (R/V) *Natsushima* cruise NT13–15 in July 2013 at a depth of 985 m (i.e., 9.9 MPa). Subsequent onshore incubation of the deep-sea sediments within the HP-Core, including liquid media and gaseous amendments, was performed for 45 days at the Kochi Core Center (KCC), Japan. We report here microbiological and geochemical results indicating a successful deployment and onshore use of the HP-Core.

At the Joetsu Knoll, massive methane hydrates outcrop on the seafloor, sourced mainly from thermogenically produced methane (Matsumoto et al., 2005). In addition to this rich source of reduced carbon, the Joetsu Knoll is bathed in oxygen-rich bottom water (>210 μmol/kg; Gamo and Horibe, 1983; Gamo, 2011), fueling diverse chemosynthetic microbial consortia. Previous 16S rRNA clone libraries from sediments at the Joetsu Knoll have revealed the presence of anaerobic methane oxidizing archaea (e.g., ANME-1 and ANME-2) in addition to other diverse Archaea and Bacteria (Yanagawa et al., 2011). Despite the high concentration of oxygen in overlying bottom waters, the presence, distribution, and/or activity of aerobic methanotrophs associated with methane hydrates has not been specifically investigated at the Joetsu Knoll. In this study, we incubated outcrop sediment samples associated with methane hydrates under the HP condition with stable isotope-labeled substrates, and then investigated the methanotorophic microbial communities in response to O_2_ addition by analyzing sequences of 16S rRNA genes and methane monooxygenase intergenic spacer region (MISA) between two methane mono-oxygenase genes (*pmoC* and *pmoA*; Tavormina et al., 2010).

## Materials and methods

### Experimental setup

**Figure 1:**
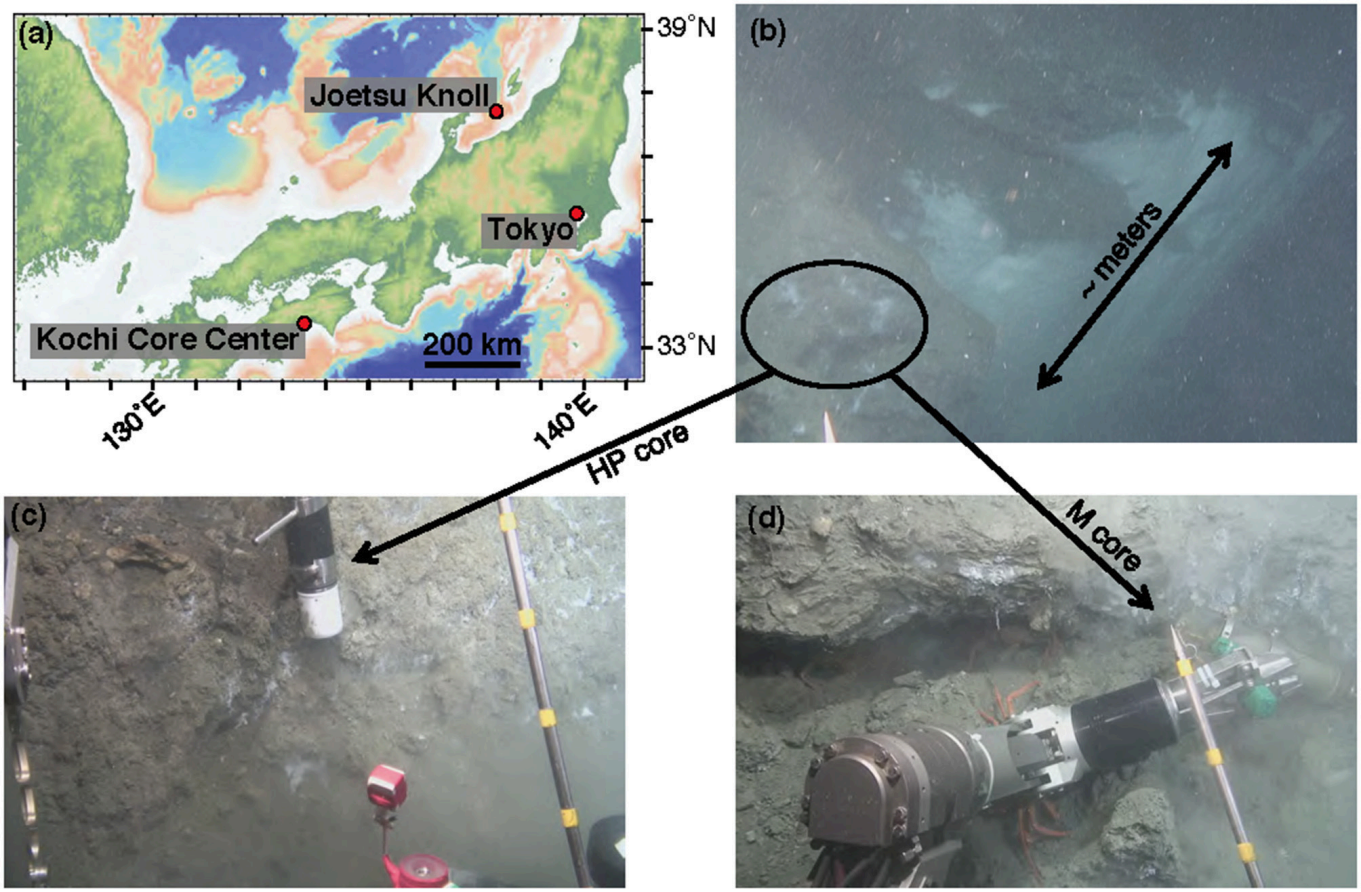
Contextualization of study site. **(a)** Map of central Japan, including the Joetsu Knoll study site. **(b)** Image capture from the ROV *Hyper-Dolphin* Dive 1555, demonstrating the sampling location along a several meter-sized wall of methane hydrate and bacterial mats. Samples were taken from roughly in the area of the black circle. **(c)** Sediment capture using the HP-Core sampler. **(d)** Sediment capture using the M-Core sampler.

**Figure 2:**
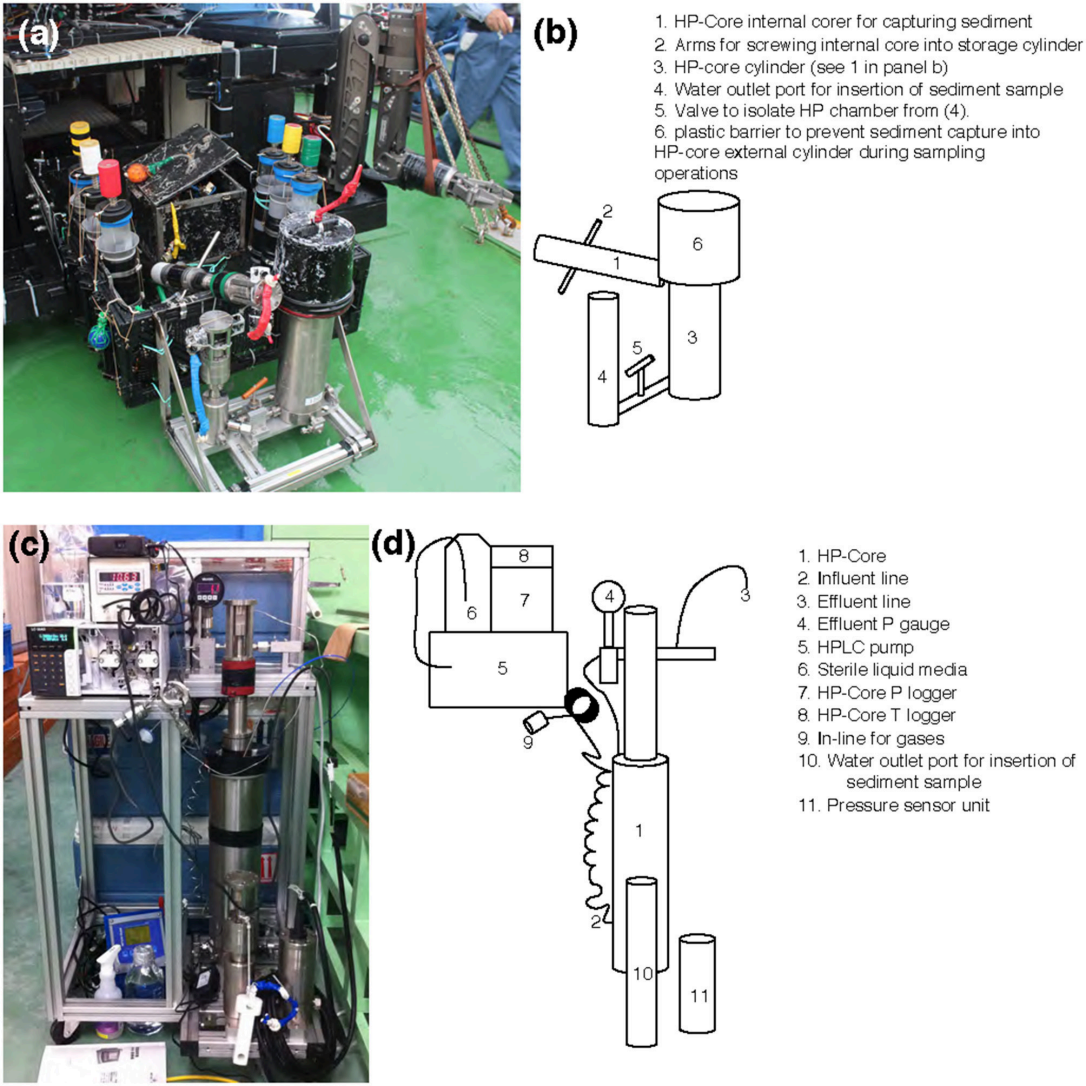
**(a,c)** Photographs and **(b,d)** schematics of the HP-Core. An aluminum chassis **(a,c)** holds the stainless-steel cylinders comprising the HP-Core. This chassis can be attached to the payload of a remotely operated vehicle (e.g., ROV *Hyper-Dolphin*) for deployment **(a)**. This does not preclude the simultaneous deployment of other sampling equipment, including push cores and collection boxes **(a)**. After recovery of the HP-Core onboard ship or onshore, the HP-Core (still supported by the aluminum chassis) is connected to pressure and temperature loggers, an HPLC pump for influent media, and an outflow hose for effluent sampling. The influent line [2] enters at the bottom of the HP-Core [1], while the effluent line [3] exits from the top of the HP-Core [1]. The HP-Core is ~0.5 m in height.

Sample collection was performed during the NT13–15 cruise aboard the R/V *Natsushima* during July 2013. The study site, the Joetsu Knoll, is a well-characterized location of methane seepage offshore the Joetsu City, Niigata Prefecture, Japan (Figure 1; 37°31.1′N, 137°58.0′E, 985 mbsl). Two methane hydrates-containing sediment cores were collected during Dive 1555 of the ROV *Hyper-Dolphin*. Sediment was collected into the HP-Core (internal volume: ~920 mL, maximum pressure to use: 50 MPa) by abrading the internal core cylinder against an exposed vertical wall of sediment interlaced with white methane hydrates (Figure 1). The HP-Core's internal cylinder (label “1” in Figure 2b) was then immediately placed into the external cylinder (label “3” in Figure 2b) and secured by tightening (using label “2” in Figure 2b). In this manner, sediment was collected at environmentally relevant pressure and sealed into the HP-Core *in situ*, in order to maintain pressure throughout core recovery and onshore experimentation (see Figure 1 for *in situ* sampling photographs; see Figure 2a for arrangement of HP-Core on the ROV *Hyper-Dolphin* payload). During this deployment, we discovered that the Teflon seal on the HP-Core was compromised, most likely by small sediment grains lodged against the Teflon core liner which resulted in a partial loss of pressure (from 9.9 Mpa down to ~2 MPa) during transit of the ROV *Hyper-Dolphin* from seafloor to the R/V *Natsushima*. The core was immediately re-pressurized up to 10 MPa onboard ship by injection of 0.2 μm-filtered artificial seawater; specifically, we dissolved the following components into 1 L of MilliQ water (Merck Millipore, Massachusetts, USA): 0.14 g KH_2_PO_4_, 0.54 g NH_4_Cl, 3.05 g MgCl_2_·6H_2_O, 0.11 g CaCl_2_, 20.45 g NaCl, and 10 mL trace element solution (1.5 g nitrilotriacetic acid, 3.0 g MgSO_4_·7H_2_O, 0.59 g MnCl_2_·4H_2_O, 1 g NaCl, 0.1 g FeSO_4_·7H_2_O, 0.18 g ZnSO_4_·7H_2_O, 0.01 g CuSO_4_·5H_2_O, 0.02 g AlK(SO_4_)_2_·12H_2_O, 0.01 g H_3_BO_3_, 0.007 g Na_2_SeO_3_·H_2_O in 1 L of MilliQ H_2_O). The HP-Core apparatus was stored at 4°C and 10 MPa onboard, during shipment, and upon arrival at the KCC laboratory. Besides the HP-Core, a second core was collected from adjacent hydrate-containing sediment into a traditional M-type corer (hereafter, “M-Core”). The material collected into the M-Core contained a mixture of sediment and bottom water, which by the time of recovery onboard ship had separated by density. Immediately onboard ship, subsamples of the “M-Core water” and “M-Core sediment” were frozen at −80°C for later DNA extraction and sequencing.

**Figure 3:**
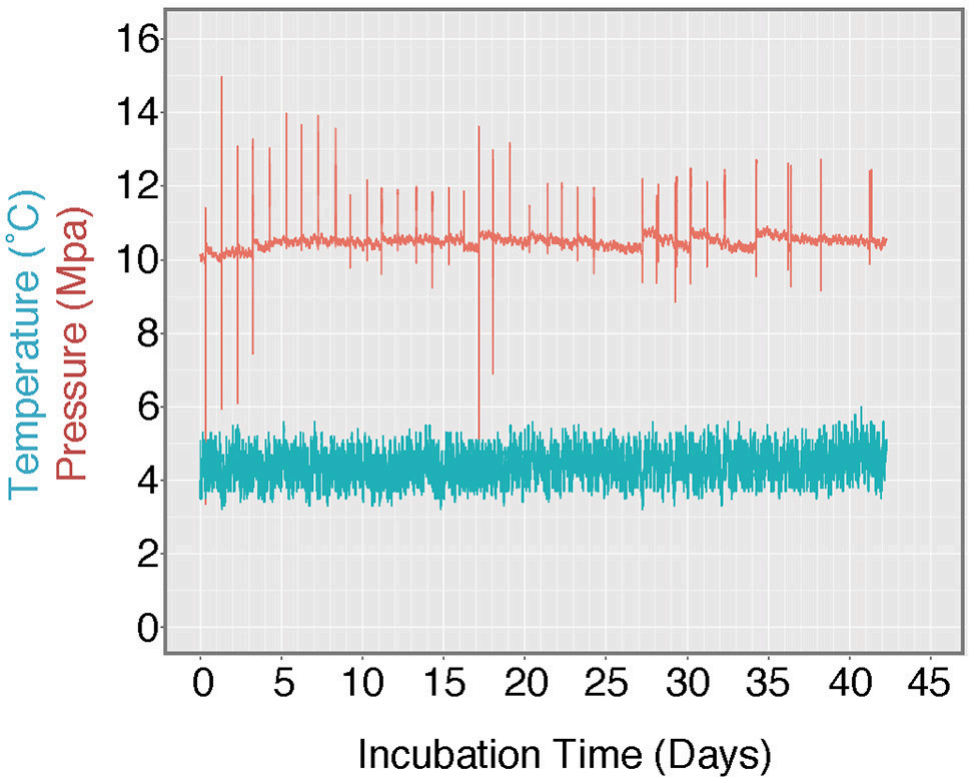
Log of HP-Core temperature and pressure over the duration of incubation. Temperature was maintained at ~4.5°C by storing the HP-Core in a walk-in refrigerator throughout the experiment. Pressure was maintained at ~10 MPa (chosen to match the environmental pressure at the sampling depth of 985 mbsl) by injection of sterile artificial seawater via modified HPLC pump. Spikes in the pressure log record the daily effluent sampling for δ^13^C_DIC_, during which time pressure fluctuated as the effluent port was opened. Over the course of >40 days, user technique improved and the fluctuations in pressure decreased in magnitude.

**Figure 4:**
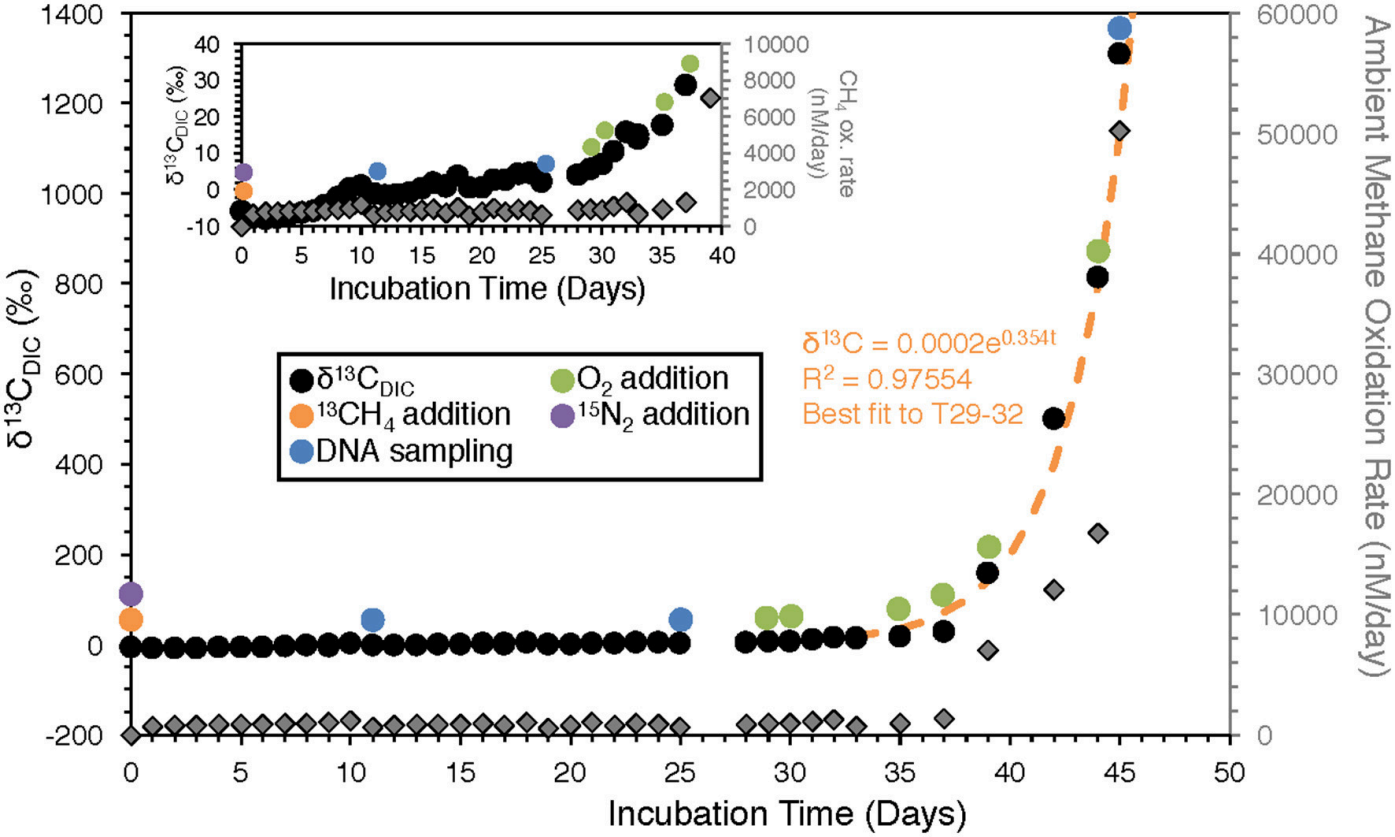
Time-resolved record of HP-Core incubation. Daily δ^13^C_DIC_ measurements are given in black circles. Colored circles represent sampling or amendments (see legend). Gray diamonds are the calculated methane oxidation rate between each day and the day prior. Inset shows the same data on a smaller y-axis in order to better resolve trends within the first 40 days of the experiment.

A unique and hence important function of the HP-Core system is the ability to supply liquid and gas substrates while maintaining the constant pressure in the chamber. In this study, the HP-Core was kept for the duration of experimentation (total 45 days) in a walk-in 4°C refrigerator in the laboratory (Figures 2c,d). Twelve days after collection from the seafloor, the HP-Core was amended with ^13^CH_4_ (50 mL of 50% ^13^CH_4_) and ^15^N_2_ (50 mL of 50% ^15^N_2_) and daily tracking of pressure, temperature, dissolved inorganic carbon concentration (DIC), and δ^13^C_DIC_ began for the course of a 45-day experiment in the HP incubation of seafloor microbial assemblages (Figures 3, 4). Temperature and pressure were continuously monitored (Δt = 1 s), with daily samples taken for δ^13^C_DIC_. During daily sampling, pressure was kept at 10 MPa by injection of sterile artificial seawater that contained no carbon sources (see Supplementary Text). Samples for microbial community analysis were taken from the sediment water slurry at 11, 25, and 45 days (hereafter, T11, T25, and T45, respectively). Samples collected during the time course experiment at T11 and T25 consisted of water from the effluent outflow at the top of the HP-Core. This involved bleeding 6 mL of effluent from the top port, followed by filtration onto a 0.2 μm-pore sized polycarbonate membrane and freezing at −80°C. The final T45 time point consisted of sediment at the bottom of the HP-Core during the takedown of the experiment. ^13^CH_4_ and ^15^N_2_ were injected at the start of the HP incubation experiment followed by periodic additions of 100% O_2_ on days 29, 30, 35, 37, 39, and 44 to stimulate aerobic methane-oxidation (Figure 4). In all cases 10 mL (at room condition) of O_2_ was injected, which corresponds to the O_2_ concentration *in situ* (~210 μmol/kg, Gamo, 2011), with the exception of the first injection on day 29, which was half this volume.

### Dissolved inorganic carbon (DIC) and δ^13^C measurement

Carbon concentration and isotopic measurements were conducted on 0.2 μm-filtered effluent water samples <24 h after collection. Measurements were performed on an isotope-monitoring gas chromatography/mass spectrometry (irm-GC/MS) Thermo Finnigan Delta Plus XP isotope-ratio mass spectrometer connected to TRACE GC as previously described (Ijiri et al., 2012).

### DNA extraction and sequencing

DNA was extracted from M-Core sediment and water samples using the MoBio PowerMax soil DNA isolation kit, according to manufacturer protocols (~5 g slurry/extraction). The T11, T25, and T45 (duplicate samples of T45 were extracted and sequenced) time points were extracted with the MoBio PowerSoil DNA isolation kit, according to manufacturer protocols (~0.5 g/extraction). In addition, duplicate T45 sediments were separately subjected to a simplified hot alkaline DNA extraction (Morono et al., 2014), in which sequential cell lysis is performed in heated 1M sodium hydroxide solution (Supplementary Text).

Samples were prepared for iTag-sequencing of the V4 region of the 16S rRNA gene, according to a slightly modified version of the Earth Microbiome Project's recommended protocol (see Mason et al., 2015 for protocol modifications). New England Biolabs Q5 polymerase enzyme was additionally substituted for 5-PRIME Hot Master Mix. Sequencing was performed on an Illumina MiSeq platform at Laragen, Inc. Culver City, CA, and data processing [joining paired ends, trimming sequences, chimera checking, 97% operational taxonomic unit (OTU)-picking, and taxonomic assignment] were performed as previously described (Case et al., 2015). Nonmetric multidimensional scaling (NMDS) analyses were performed in the R environment using the “vegan” package on square-root-transformed tables of relative sequence abundance (Oksanen et al., 2013; R Core Team, 2014).

In addition to sequencing of 16S rRNA genes, an assay of the monooxygenase intergenic spacer region (“MISA”) between two methane mono-oxygenase genes (*pmoC* and *pmoA*) was performed following previously described protocols (Tavormina et al., 2010; see Supplementary Text for modified primer sequences) on two samples: M-Core sediment and T45 sediment. Transformation of the MISA fragment into *E. coli* was performed with the 10G Elite Solo kit (Lucigen Inc., Culver City, CA). Inserts were amplified with the Lucigen Corporation GC Vector Amplification pSMART kit and separately digested with *Hae* III and *Rsa* I restriction enzymes in order to generate restriction fragment length polymorphism (RFLP) patterns. Unique inserts were sequenced at Laragen, Inc. The resulting traces were manually checked for quality, translated to amino acid sequences, aligned against pure culture and previously published *pmoA* fragments in MUSCLE (Edgar, 2004), and trimmed to the *pmoA* amino acid positions 5–49 of *M. capsulatas Bath* (an approach employed in Tavormina et al., 2010). These *pmoA* fragments, both experimental and from known organisms, were used to generate a 100-bootstrap, maximum likelihood tree in RAxML (Stamatakis, 2014).

### DNA accession numbers

The 16S rRNA gene sequence data have been submitted to the NCBI BioProject database under the BioProject ID of PRJNA416818. The *pmoA* and *pmoC* sequence data are available in the GenBank database with the accession numbers of MG149702-MG149775.

## Results

### High-pressure sediment core sampler

Of the five goals planned for the first deep-sea deployment of the HP-Coring device, all were successful, with the exception of partial depressurization during core recovery. The first goal, to develop a HP-Core deployable on the submersible payload, was achieved by deployment with the ROV *Hyper-Dolphin*. Furthermore, the relatively small HP-Core footprint enabled simultaneous deployment of other sampling devices during the dive [e.g., 6 push cores, a plastic tote for recovery of push cores previously deployed on the seafloor, a temperature probe and deep-sea high-pressure CO_2_ injection system (Ohtomo et al., 2015), and the “M-type” corer], demonstrating that use of the HP-Coring device can be incorporated with other ROV-based scientific objectives and does not require an exclusive deployment.

Our second goal was to collect a core >10 cm in length using the HP-Core. Due to the nature of the hydrate environment at the Joetsu Knoll, we did not deploy the HP-Core like a traditional vertical push core at the seabed, we instead abraded the HP-Core against a wall of methane hydrate interspersed with sediments and bacterial mats. Abrading the HP-Core against the wall of hydrate and sediment allowed us to visualize the glass-made corer during sampling and gauge how the device was responding during the first deployment of this new technology. Using this approach enabled recovery of a significant amount of sediment, similar to the amount captured in a traditional push core.

Unfortunately, the third technical goal, to maintain *in situ* HP conditions through recovery onboard ship, was not met during the NT13–15 cruise. The HP-Core arrived at the sea surface having lost ~8 MPa of pressure down to ~2 MPa, believed to be the result of small sediment grains that compromised the Teflon seal where the core interfaces with the core liner. Pressure was immediately restored with filtered seawater upon recovery, and held stably during incubation at sea, transport and over the course of the 45 days-experiment in the lab, demonstrating its resilience to shipping and handling.

Goals four and five were to demonstrate the addition of liquid and gas amendments to the incubator under pressure during the HP-incubation experiment, and to be capable of extracting time-resolved output samples. Successful sampling from the outflow port for geochemical analysis was performed daily. Sampling resulted in a minor loss of internal pressure (generally <1 MPa, dependent on the user's skill level; Figure 3). Pressure was restored after sampling by pumping in fresh, sterile artificial seawater. Additionally, gas-phase amendments were injected in-line with seawater replacement throughout the incubation.

### Geochemical results

Over the course of 45 days, δ^13^C_DIC_ was observed to increase after ^13^CH_4_ addition. The first ~30 days showed slow progressive enrichment ^13^C_DIC_ (Figure 4; for DIC concentration, see Supplementary Figure 1). A model of exponential increase in daily δ^13^C_DIC_ values fit from the data between T29 and T32 (*R*^2^ = 0.97; Figure 4), matches the data from the later time interval T33–T45 very well; and this increase appears to be associated with the direct injection of O_2_ beginning at T29. Stepwise rates of methane oxidation at 10 MPa were calculated by subtracting the moles of ^13^C observed between time points (t_n_-t_n−1_), based on the assumption that new ^13^C in the DIC pool represented newly oxidized ^13^CH_4_ (Equation 1; a factor of 2 was added because the methane amendment was only 50% ^13^CH_4_). The trend in methane oxidation rate, by definition, mirrors the increase in δ^13^C_DIC_ and shows increasing rates of methane oxidation late in the HP-incubation experiment.

(1) $$\begin{array}{l} {\text{CH}}_{4}\text{ oxidation rate }(\text{nM}/\text{day})\\ \;=2\;•\;10^{6}\;•\;[\left({\left[\text{DIC}\right]}_{\text{n}}\;•\;{\text{V}}_{\text{HP}-\text{Core}}\right)\\ \;-({\left[\text{DIC}\right]}_{\text{n}}/\left(1+{\text{R}}_{\text{std}}((\delta^{13}{\text{C}}_{\text{n}}/1000)+1))\right)\\ \;-({\left[\text{DIC}\right]}_{\text{n}-1}/\left(1+{\text{R}}_{\text{std}}((\delta^{13}{\text{C}}_{\text{n}-1}/1000)+1))\right)]/(\text{t}-{\text{t}}_{-1}) \end{array}$$

Within the initial period of the experiment prior to rapid increase in methane oxidation rate (T0–T29), slow but measurable methane oxidation was observed (c.f. inset of Figure 4), with higher rates (increase in δ^13^C) over the first 10 days relative to days T11–T29.

### Microbial diversity analysis

**Figure 5:**
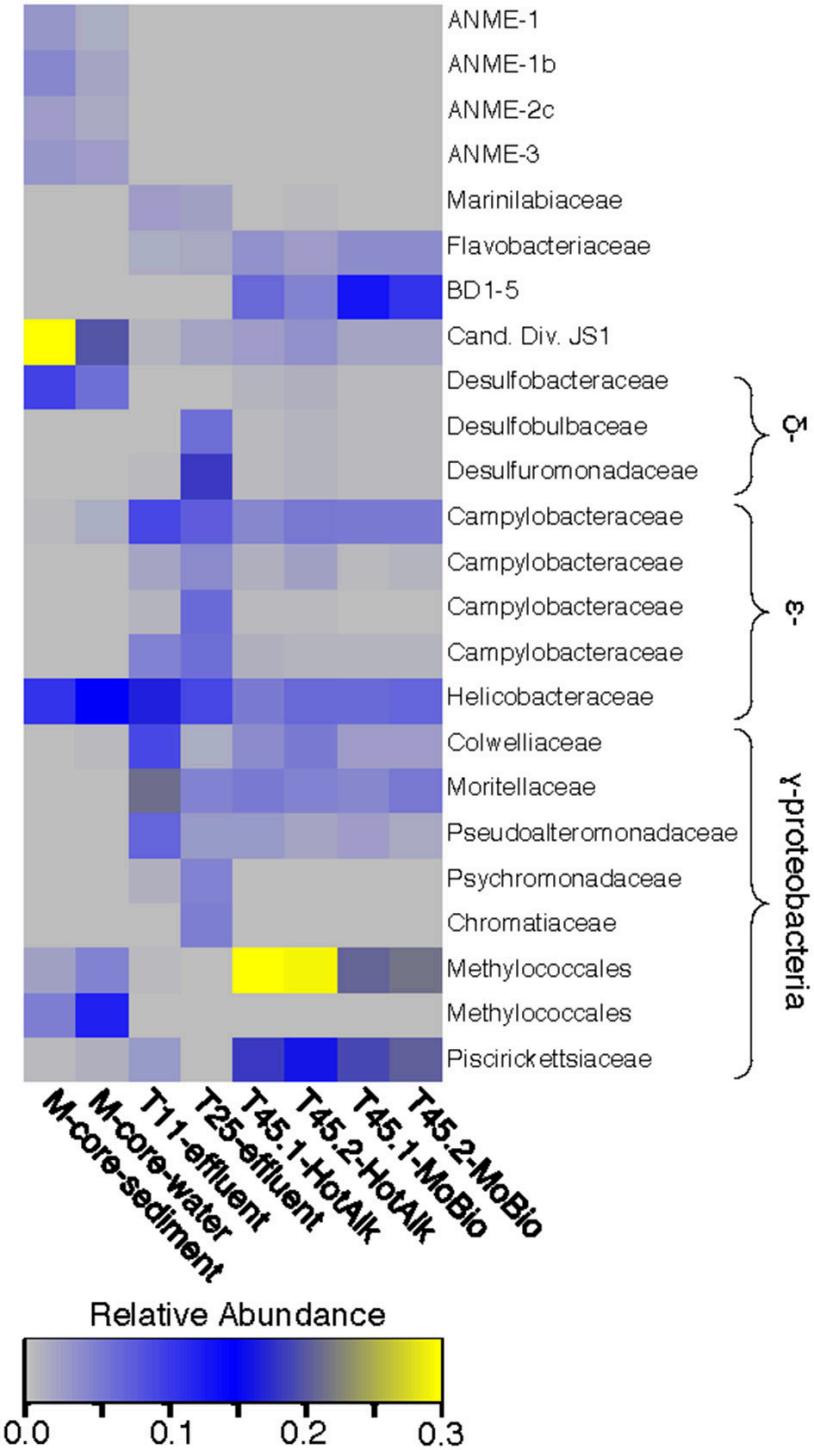
Heat map of major OTUs identified in the 16S rRNA gene iTag-sequence dataset. OTUs were only selected for presentation if they were present at >2% relative abundance in the M-Core, HP-Core-effluent (T11 and T25), or HP-Core-sediment (T45) samples. M-Core samples are characterized by their richness in Candidate Division JS1 bacteria. T11 and T25 effluent samples host a wide diversity of Delta-, Epsilon-, and Gammaproteobacteria, but notably differ from the T45 samples which are rich in a *Methylococcales*-associated OTU. The full table of 16S rRNA gene data is provided in the Supplementary Data.

**Figure 6:**
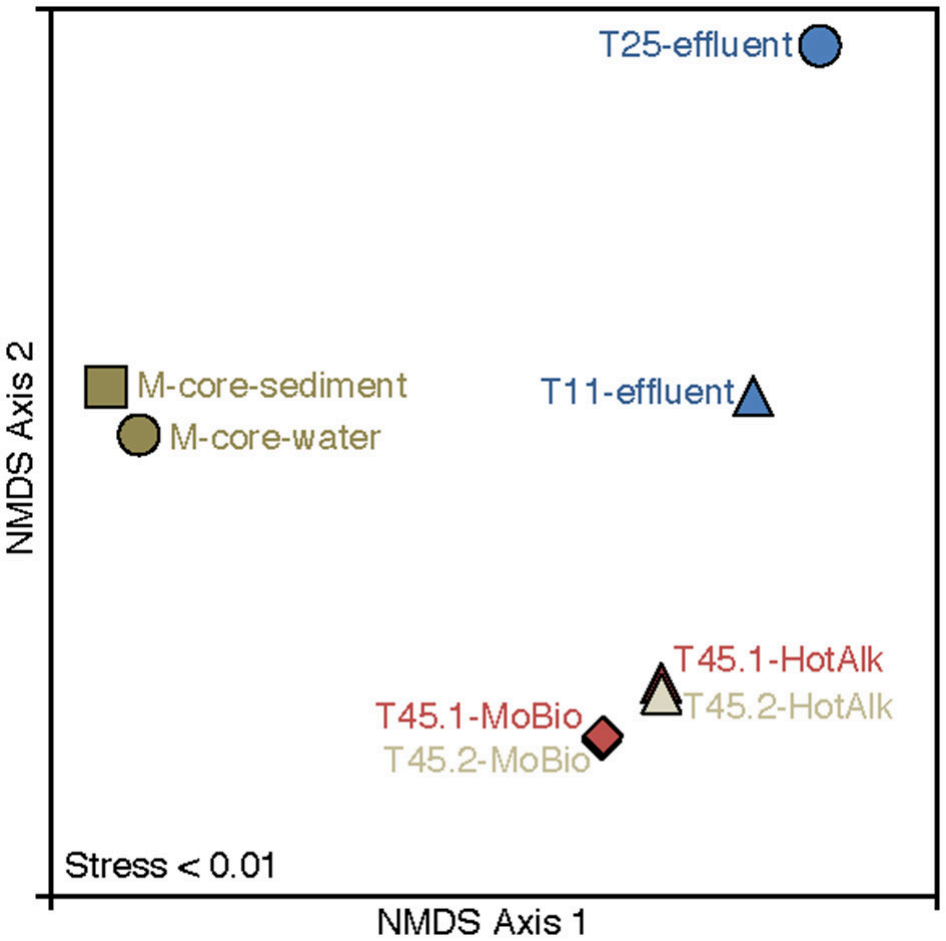
Nonmetric multidimensional scaling (NMDS) plot of 16S rRNA iTag-sequence data from this study. The microbial communities in the samples naturally break into three categories: M-Core sediments, HP-Core effluent, and HP-Core sediments. Among the HP-Core sediments, DNA extraction method accounts for a measurable but small difference in recovered microbial community composition.

The microbial diversity was analyzed by 16S rRNA iTag-sequencing from the background M-core, collected adjacent to the HP-core, and from different time points in the HP-Core incubation, including effluent samples from T11 and T25, and the final HP-Core sediment samples from T45 (Figures 5, 6). The M-core samples, including both sediment and overlying water, were characterized by high relative abundances of OTUs-associated with Candidate Division JS1 bacteria (20–30%), *Desulfobacteraceae* (6–10%), *Methylococcales* (2–12%), and ANME archaea (1–4%); (Figure 5; Supplementary Data). Analysis of the liquid effluent samples sampled from the pressure incubation experiment during T11 and T25 did not detect OTUs associated with ANME archaea or *Methylococcales*. Instead, these samples showed high relative abundance of *Helicobacteraceae* (9–17%), as well as other OTU's associated with Epsilon-, Delta-, and Gammaproteobacteria and Bacteroidetes (Figure 5). Sediment samples collected at the termination of the HP-core incubation (T45) showed harbored much of the same diversity of Epsilon-, Delta-, and Gammaproteobacteria observed in the HP-Core effluent samples, with the exception of the recovery of JS1 OTUs (2–4%) and a high percentage of *Methylococcales*. had a high relative abundance of OTU's associated with *Pisirickettsiaceae* (16–20%), which was a minor component of the M-Core and HP-Core effluent samples (Figure 5). The extraction method (MoBio power soil kit vs. Hot Alkaline lysis) appears to contribute a minor difference in the overall microbial 16S rRNA signature recovered from T45 sediments. When extracted with the MoBio kit, a *Methylococcales*-associated OTU is recovered at about two-thirds the relative abundance as recovered in samples extracted with the Hot Alkaline method. In contrast, a BD1-5-associated OTU is twice as abundant in MoBio-extracted samples as compared to Hot Alkaline-extracted samples (Figure 5). These differences are apparent in multidimensional ordination, where the T45 samples overall plot closely together, but are distinctly separate according to extraction method (Figure 6). This variation may be due to differential lysis, as has been reported in other studies (Morono et al., 2014).

**Figure 7:**
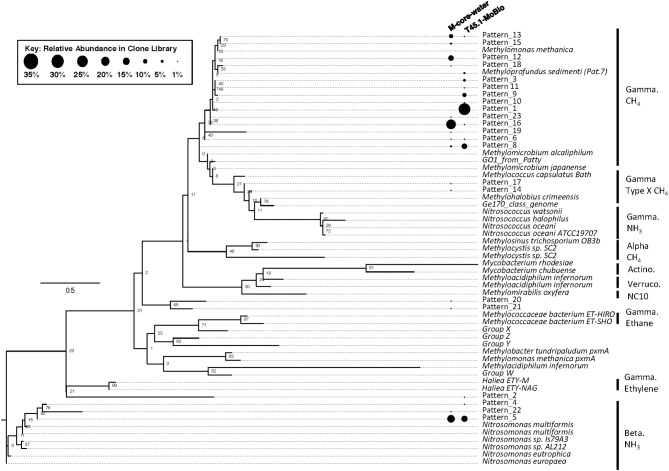
Maximum likelihood tree of *pmoA* gene sequences generated in the MISA assay (trimmed to amino acids 5–49 of *M. capsulatas Bath*) from the M-Core and HP-Core T45 incubation. Sequences from this study are defined as “Pattern_*x*” according to unique *Hae* III and *Rsa* I-digested RFLP profiles. Sequences from cultured organisms and sequenced genomes of methane- and ammonia-oxidizers are given with their species name. Labels denote the phylogenetic groupings and predicted oxidation metabolisms. Multiple sequence alignments were generated in MUSCLE and the tree was generated in RAxML with 100 bootstraps. Black circles represent relative abundance of *pmoA* sequences in the M-Core water and T45.1-MoBio samples. The largest contrast between the two samples is seen in the abundance of different methane-oxidizing Gammaproteobaceria-affiliated *pmoA* patterns.

Analysis of putative methanotrophic bacterial diversity was also assessed using the particulate monooxygenase-targeted MISA assay targeting both particulate methane monooxyganse (*pmoA*) and ammonium monooxygenase (*amoA*) genes (Tavormina et al., 2010). Using the MISA assay, we screened the overlying water from the M-Core and T45.1-MoBio sediment, and detected diverse *pmoA* sequences with a lower proportion of *amoA* sequences, with some overlap in MISA fragments recovered between the *in situ* M-Core and HP-Core after the 45-day incubation. Both samples showed a high relative abundance of a *pmoA* sequence putatively associated with gammaproteobacterial methanotrophs (Patterns 1 and 16 in Figure 7). Overall the *pmoA* sequences recovered in both samples were more similar to one another than to other *pmoA* genes reported from other sites or from cultured organisms (Figure 7).

## Discussion

The HP-Core was successfully deployed on the payload of the ROV *Hyper-Dolphin* during Dive 1555, capturing sediment in a challenging deep-sea environment and retaining methane hydrate-associated sediment within the reaction chamber through recovery onboard ship. The loss of partial pressure during recovery was unfortunate; however, the HP-Core successfully maintained high pressure through shipping and during the course of 45 days of experimentation. There is no indication that the HP-Core would not have continued to retain pressure for a significantly longer experimental duration. To prevent the partial pressure loss during the core recovery, the back-up pressure function (e.g., N_2_ reservoir that supports the pressure at a pre-defined value as an accumulator; see Kubo et al., 2014) would be an option.

Based on our experience with this first deployment, when deploying this first generation of the HP-Core, we recommend choosing carefully the sampling site and being cautious to avoid unnecessary sediment disturbance—excess sediment clouding bottom waters increases the likelihood of a compromised Teflon seal. Depending on time constraints, it is advisable to choose a seafloor location, hold the vehicle steady for enough time to let particles settle out, and only then to perform sampling. Additionally, it is good practice to perform HP-Core sampling as the last function of a deep-sea dive. This minimizes both the amount of jostling on the HP-Core and the time duration between sampling and recovery onboard ship.

Some recommendations can be made for future iterations of the technical design for the HP-Core. The first generation had one outflow sampling port, located at the top of the vessel. This port worked well, but daily outflow samples only represented the suspended microbial community at the top of the incubation. Based on our 16S rRNA sequencing data (Figures 5, 6), we suspect this resulted in the different microbial community observed between the suspended slurry and microorganisms primarily associated with sediments settled at the bottom of the column. Future iterations of the HP-Core would be improved by having multiple outflow ports located at various heights along the incubation column. Similarly, the inflow port for adding liquid media and gas amendments only existed at the bottom of the incubation column. Although this worked for our experimental design, it is conceivable that future experiments would benefit from an ability to add amendments from either the top or bottom of the chamber–requiring engineering of additional inflow ports in future designs.

### Microbially mediated methane oxidation during HP incubation with ^13^CH_4_

During the 45-day incubation, DIC and δ^13^C_DIC_ data (collected and analyzed daily) suggested methane oxidation (Figure 4). In the first 11 days (T0–T11) of the experiment methane oxidation, as determined by incorporation of ^13^C into the DIC pool, appeared to be accelerating. However, it then plateaued and between T11 and T28 little methane oxidation was observed. We suspect the methane oxidation at the start of the incubation was associated with aerobic methanotrophy which ceased when O_2_ was fully consumed. The theoretical amount of O_2_ consumed by aerobic methanotrophy can be calculated by stoichiometric conversion using the following equation and ^13^C_DIC_ data to track the number of moles of methane consumed between T0 and T11:

(2) $$\begin{array}{l} \text{C}{\text{H}}_{4}+2{\text{O}}_{2}⇌\text{C}{\text{O}}_{2}+2{\text{H}}_{2}\text{O} \end{array}$$

With 4.25 μmol/kg of CH_4_ consumed between T0 and T11 (calculated from data in Figure 4), corresponding oxidation of 8.50 μmol/kg of O_2_ is required. This is a relatively small amount compared to known bottom water O_2_ concentrations in the deep-sea (>220 μmol/kg), but it is likely that through the course of shipment of the HP-Core to KCC and static storage for 12 days at 4°C prior to ^13^CH_4_ addition, oxygen levels may have been depleted in the incubation chamber relative to *in situ* concentrations from aerobic respiration (e.g., via sulfide oxidation or organic carbon) Respiratory processes could have continued during T0–T11, all independent of ^13^C-label and thus undetected by our geochemical measurements.

Between T11 and T28, the rate of methane oxidation was slower, with 7 μmol/kg of CH_4_ oxidized during the 7 days. If we assume that during this period methane-oxidation there was a shift to anaerobic oxidation of methane coupled to sulfate (AOM; Equation 3), then only 7 μmol/kg of sulfate are stoichiometrically required:

(3) $$\begin{array}{l} \text{C}{\text{H}}_{4}+\text{S}{\text{O}}_{4}^{2-}⇌\text{H}{\text{S}}^{-}+\text{HC}{\text{O}}_{3}^{-}+{\text{H}}_{2}\text{O} \end{array}$$

This is well within the bounds of seawater chemistry, where sulfate is present at ~28 mmol/kg. Anaerobic conditions during this period were supported by oxidation-reduction potential (ORP) measurements. When the first ORP measurement was taken, at T29, it was at the very reduced value of −300 mV. Although we do not have ORP data to help define exactly when anaerobic conditions began during the incubation, it is clear that by T29 anaerobic conditions prevailed.

The apparent rapid switch to aerobic methanotrophy in the high-pressure incubation was triggered by the addition of oxygen at T29, stimulating an exponential rise in δ^13^C_DIC_ shortly after injection. Over the course of six oxygen injections between T29 and T45, 55 mL of 100% O_2_ were added to the incubation chamber, corresponding to 242 μmol/kg of O_2_. Conversion of δ^13^C_DIC_ into consumption of methane, results in an estimated consumption of 143 μmol/kg of O_2_ from aerobic methanotrophy between T29 and T45, within the range of the injected O_2_. Regular ORP measurements between T29 and T45 reflected the addition of oxygen but also its rapid consumption: after T30, ORP averaged −33 mV (max = 67 mV, min = −120 mV).

The hypothesis of sequential aerobic, anaerobic, and aerobic phases in the HP-Core incubation chamber are additionally supported by sequencing data of the 16S rRNA and *pmoA* genes (Figures 5–**7**). M-core samples, taken from sediments nearby the sampling location for the HP-Core at the Joetsu Knoll, revealed the presence of diverse anaerobic methanotrophs belonging to ANME-1, -2, and -3 as well as aerobic methanotrophs of the gammaproteobacterial *Methylococcales* order (Figure 5). The presence of aerobic methanotrophs in the M-Core, and presumably in the neighboring HP-Core at the time of collection, is further supported by recovery of *pmoA* genes related to gammaproteobacterial methane oxidizers (see Figure 7; Inagaki et al., 2004b; Tavormina et al., 2010). In addition, the microbial diversity observed in the HP-Core incubation at T45 suggests ingrowth of aerobic methanotrophs in the HP-incubated sediments amended with oxygen by the end of experimentation, with relatively abundant *Methylococcales* and loss of OTUs associated with ANME archaea (Figure 5).

In addition, the incubated T45 sediments were also enriched in other putative aerobic gammaproteobacteria (e.g., *Colwelliaceae*) and epsilonproteobacteria (e.g., *Campylobacteraceae*) OTUs than the M-core sediment. These OTUs were also observed at high relative abundance in the T11 and T25 effluent samples, suggesting they grew up during the course of the high-pressure incubation (Figure 5). Members of the *Colwelliaceae* are known piezophiles (Kusube et al., 2017) and *in situ* experiments in the deep-sea suggest they increase in abundance in response to environmental perturbation (Case et al., 2015). Similarly, many members of the epsilonproteobacteria recovered in the HP-Core incubation (*Sulfurimonas* and *Sulfurovum* spp.) are related to hydrogen or sulfide-oxidizers within the *Campylobacteriales* (Inagaki et al., 2003, 2004a; Campbell et al., 2006; see Supplementary Data). As oxygen was depleted in the HP-Core incubation chamber, sulfate would have been used as an electron acceptor, producing sulfide (both in AOM and other anaerobic respiratory processes). This might explain the increased abundance of epsilonproteobacteria-associated OTUs at T11 onward.

### Comparison of methane oxidation rates measured in the HP incubation to previously published rates

**Figure 8:**
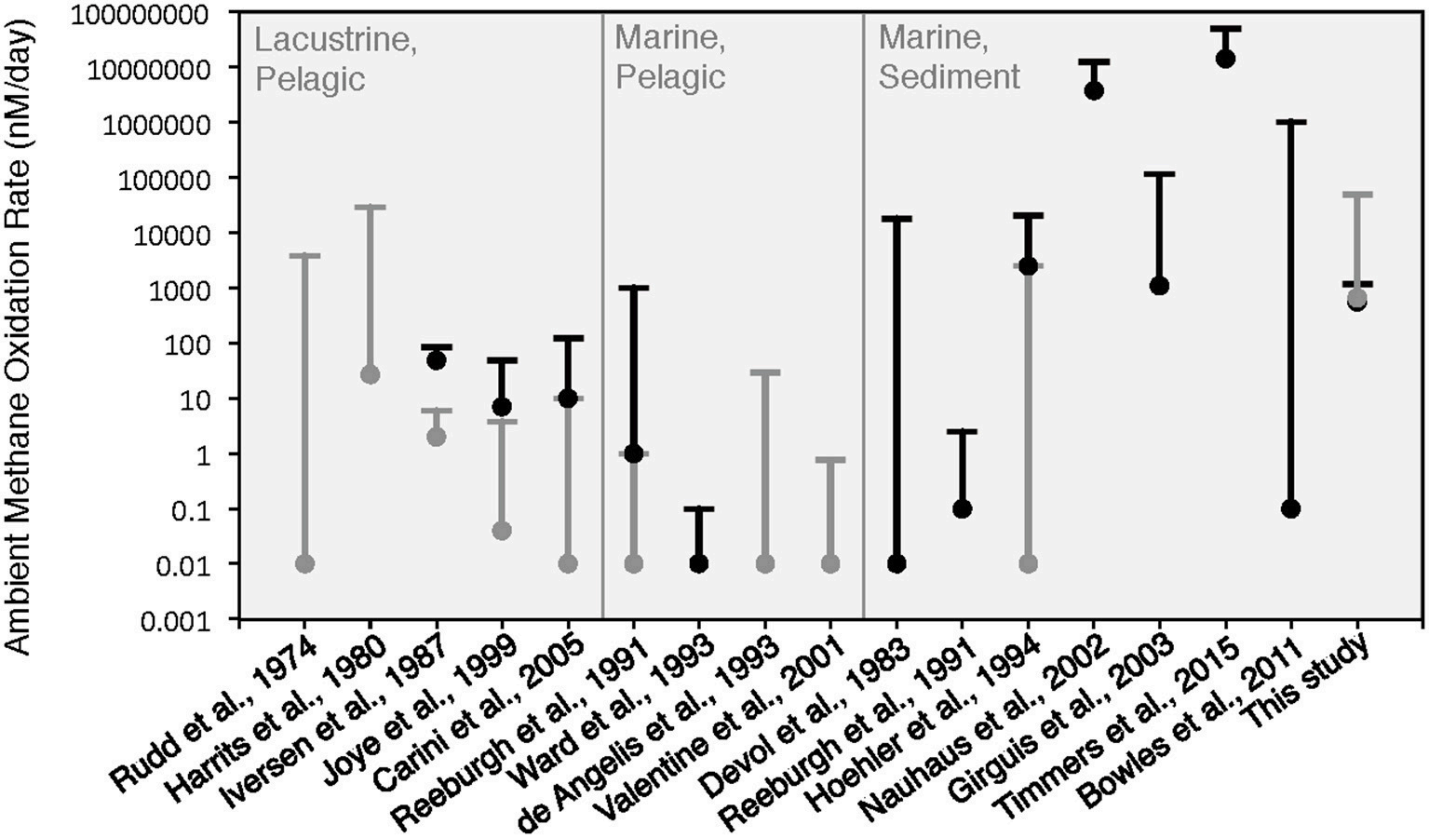
Comparison of ambient methane oxidation rate measurements between this study and previous studies. Two values are given for this study: calculated methane oxidation rates for the period of putative aerobic conditions (T0–T10 and T29–T45; in gray) and putative anaerobic conditions (T11–T28; in black). The majority of these experiments were conducted near atmospheric pressure with the exception of Nauhaus et al. (2002), which reported a notable increase in the rate of anaerobic methanotrophy with 1.1 MPa.

The rates of anaerobic methane oxidation calculated from our HP-incubation experiment are consistent with previously published rates of anaerobic methane oxidation (Figure 8). Methane oxidation rates are often observed to vary by many orders of magnitude, depending on the site, methane flux and electron acceptor concentrations (Rudd et al., 1974; Harrits and Hanson, 1980; Devol, 1983; Iversen et al., 1987; Reeburgh et al., 1991; De Angelis and Lilley, 1993; Ward and Kilpatrick, 1993; Hoehler et al., 1994; Joye et al., 1999; Valentine et al., 2001; Nauhaus et al., 2002; Girguis et al., 2003; Carini et al., 2005; Bowles et al., 2011; Boetius and Wenzhöfer, 2013; Timmers et al., 2015). Our ^13^C_DIC_ derived rates of methane oxidation in the HP-Core incubation between T11 and T29 (presumed to be primarily attributed to sulfate coupled-AOM) were low compared to measurements from other high pressure deep-sea seep incubations (Nauhaus et al., 2002; Timmers et al., 2015), and more similar to rates reported from coastal systems (Hoehler et al., 1994) or methane seep sediments with widely measured rates (Bowles et al., 2011). Rates of aerobic methanotrophy measured in the HP-Core incubation at 10 MPa were typically elevated above the majority of previously published rates from marine sediments (Figure 8). The pressure effects on aerobic methanotrophy has not been studied in detail; however, the periodic addition of oxygen to the HP-incubation system stimulated methanotrophic activity and an increase in gammaproteobacterial methanotrophs.

## Conclusion

We have presented microbiological and geochemical data representing the first successful *in situ* deployment of a new HP-Core sampler, enabling deep-sea sediment core collection and long-term maintenance of samples under high pressure. This study highlights the functionality of the HP-Core incubation system for stable isotope-labeling experiments, with *in situ* sampling of a ~1,000 m-deep methane hydrate-bearing sedimentary outcrop and water from the Joetsu Knoll, Japan. Future iterations of the HP-Core design will incorporate improvements for sampling (e.g., multiple effluent outflow ports, back-up accumulator), and *in situ* deployment of the HP-Core will hopefully increase over time as multiple laboratory groups gain access to the technology. The extent to which pressure effects the physiology of deep-sea microorganisms and rates of biogeochemical cycles continues to be an important area of research, one hopefully made more accessible to researchers through the emergence of new technologies like the HP-Core designed for direct seafloor sampling and time resolved, long-term incubation.

## Author contributions

FI and VO: designed the research; FI: led the NT13–15 cruise; YM and FI: designed the high-pressure sampling and incubation system; DC, AI, YM, and FI: collected the deep-sea samples; DC, AI, and YM: performed a high-pressure incubation experiment; AI: performed geochemical analyses; DC, PT, and VO performed molecular analyses; DC and AI: wrote the manuscript with significant input from FI and VO; All authors contributed to interpretation of data.

### Conflict of interest statement

The authors declare that the research was conducted in the absence of any commercial or financial relationships that could be construed as a potential conflict of interest.
