# Supplementary material for: Aerobic and Anaerobic Methanotrophic Communities Associated with Methane Hydrates Exposed on the Seafloor: A High-Pressure Sampling and Stable Isotope-Incubation Experiment
Source: Front Microbiol. 2017 Dec 19;8:2569. doi: 10.3389/fmicb.2017.02569 (PMC5742206; doi:10.3389/fmicb.2017.02569)
Supplement: Supplementary file 1 [file DataSheet1.PDF]

Supplementary Material

Aerobic and anaerobic methanotrophic communities associated with methane hydrates exposed on the seafloor: A high-pressure sampling and stable isotope-incubation experiment

David H. Case, Akira Ijiri, Yuki Morono, Patricia Tavormina, Victoria J. Orphan, and Fumio Inagaki*

*** Correspondence:** Corresponding Author: inagaki@jamstec.go.jp

1 Supplementary Text

1.1 Composition of artificial seawater used to pressurize HP Core daily

The artificial seawater (ASW) was made by reference to the composition of *Methanosarchina* sp. Strain BT-MS1, without the addition of yeast extract or any carbon source. Thee trace element solution used was not that for *Methanosarchina* spp. but *Methanothermococcus okinawensis*, because it was hypothesized that a trace element solution including $CuSO_4$ may better stimulate methanotrophy in our incubation. After all components were dissolved, the ASW was autoclaved.

1.2 Specific protocol for hot alkaline DNA extraction

The protocol is modified after (Morono et al., 2014). Recipes for lysis and neutralization buffer can be found in the original publication.

1. Pre-warm water baths to 50˚C and 70˚C.
2. Add 50 µL of lysis solution to ~50 mg sediment in PCR tube.
3. Heat for 20 min at 50˚C.
4. Centrifuge in mini-fuge for 30 sec at 25˚C.
5. Transfer supernatant to new PCR tube pre-loaded with 37.5 µL of neutralization buffer.
6. Wash remaining sample with 50 µL of 50˚C water.
7. Centrifuge in mini-fuge for 30 sec at 25˚C.
8. Transfer supernatant to the PCR tube from step (5).
9. Add 50 µL of lysis solution to remaining sediment in PCR tube.
10. Heat for 20 min at 70˚C.
11. Centrifuge in mini-fuge for 30 sec at 25˚C.
12. Transfer supernatant to new PCR tube pre-loaded with 37.5 µL of neutralization buffer.
13. Wash remaining sample with 50 µL of 70˚C water.
14. Centrifuge in mini-fuge for 30 sec at 25˚C.
15. Transfer supernatant to the PCR tube from step (12).
16. Combine supernatant from (8) and (15) into one tube.

1.3 Primers used for MISA assay

The MISA assay involves two PCR steps, both targeting the *pmoC-pmoA* intergenic spacer region. Since the original MISA publication in 2010 (Tavormina et al., 2010), the primers have been further modified. The primers employed in this study were:

```
PCR#1, Forward primer, spacer_pmoC_599f:       AAY GAR TGG GGH CAY RCB TTC

PCR#1, Reverse primer, spacer_pmoA_192r:       TCD GMC CAR AAR TCC CAR TC

PCR#2, Forward primer, spacer_pmoC626_mod_f:   RCB TTC TGG HTB ATG GAA GA

PCR#2, Reverse primer, spacer_pmoA_189r:       CCA RAA RTC CCA RTC NCC
```

2 Supplementary Figure

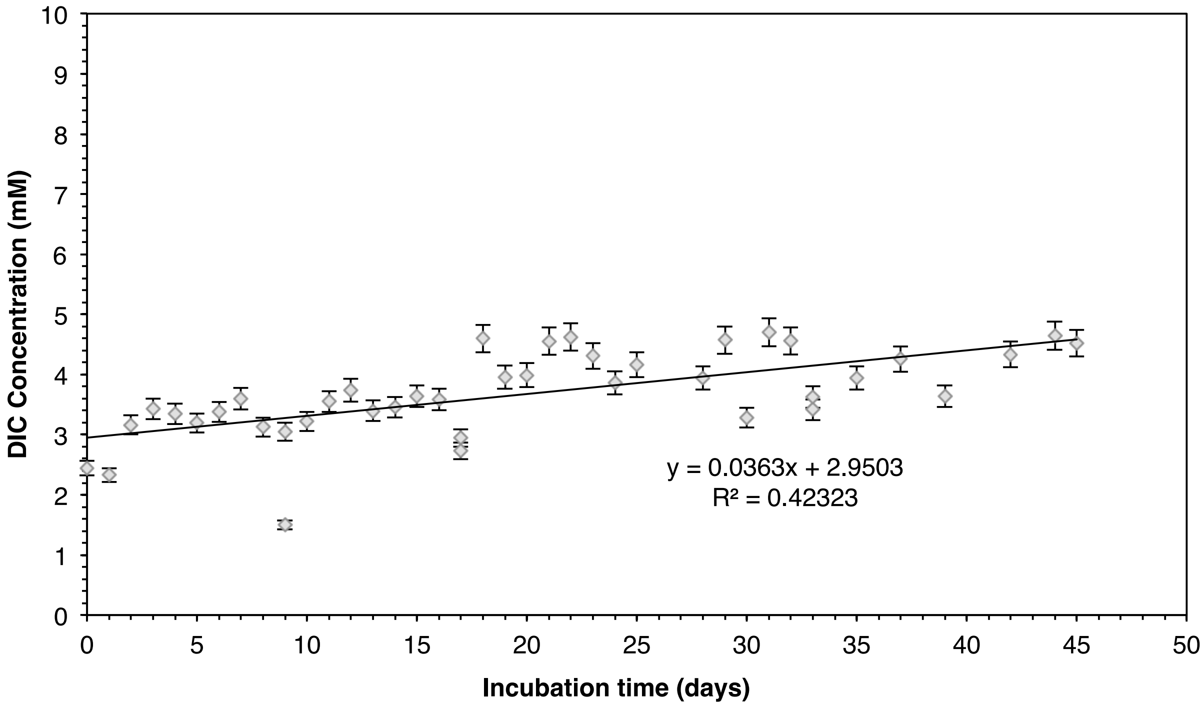

Supplementary Figure 1. Time-course measurements of dissolved inorganic carbon (DIC) concentration during the 45-day incubation. Significant variability is probably due to off-gassing of pressurized CO_2 during sampling, in which vessel fluids were taken from 10 MPa to 0.1 MPa pressure.

3 Supplementary References

Morono, Y., Terada, T., Hoshino, T., Inagaki, F. (2014) Hot-alkaline DNA extraction method for deep-subseafloor archaeal communities. *Appl. Environ. Microbiol.* **80**:1985–1994. doi:10.1128/AEM.04150-13

Tavormina, P., Ussler, W., Joye, S., Harrison, B., Orphan, V. (2010) Distributions of putative aerobic methanotrophs in diverse pelagic marine environments. *ISME J.* **4**: 700–710. doi:doi:10.1038/ismej.2009.155
